# CRISPR-enhanced microalgae biosynthesis: a promising approach for future functional feed ingredients

**DOI:** 10.1186/s40104-026-01367-1

**Published:** 2026-03-20

**Authors:** Zhen Li, Cong Li, Huan Liu, Yihao Wang, Yahui Sun, De-Xing Hou, Jianhua He, Shusong Wu

**Affiliations:** 1 Yuelushan Laboratory, Hunan Agricultural University, Changsha, 410128 China; 2 School of Energy and Mechanical Engineering, Nanjing Normal University, Nanjing, 210023 China; 3 Department of Food Science and Biotechnology, Faculty of Agriculture, Kagoshima University, Kagoshima, 890-0065 Japan

**Keywords:** Biosynthesis, CRISPR, Feed, Genome editing, Microalgae, Sustainability

## Abstract

Microalgae are photosynthetic microorganisms capable of synthesizing diverse high-value bioactive compounds, including premium proteins, polyunsaturated fatty acids, pigments, and vitamins. These natural products exhibit significant potential in enhancing livestock growth and health, offering biological activity and nutritional benefits that surpass chemically synthesized alternatives. Nevertheless, the commercial production of microalgae-derived natural products remains insufficient to meet escalating market demands. Utilizing synthetic biology strategies, especially the CRISPR system, to increase productivity of microalgae cell factories is crucial for scaling up high-value product biosynthesis. This article reviews the current applications, construction strategies, and critical pathway nodes in microalgae cell factory, with emphasis on CRISPR-based genome editing breakthroughs for optimizing microalgae nutritional profiles, and recent progress in microalgae utilization for livestock production, providing a forward-looking perspective on future developments.

**Graphical Abstract:**

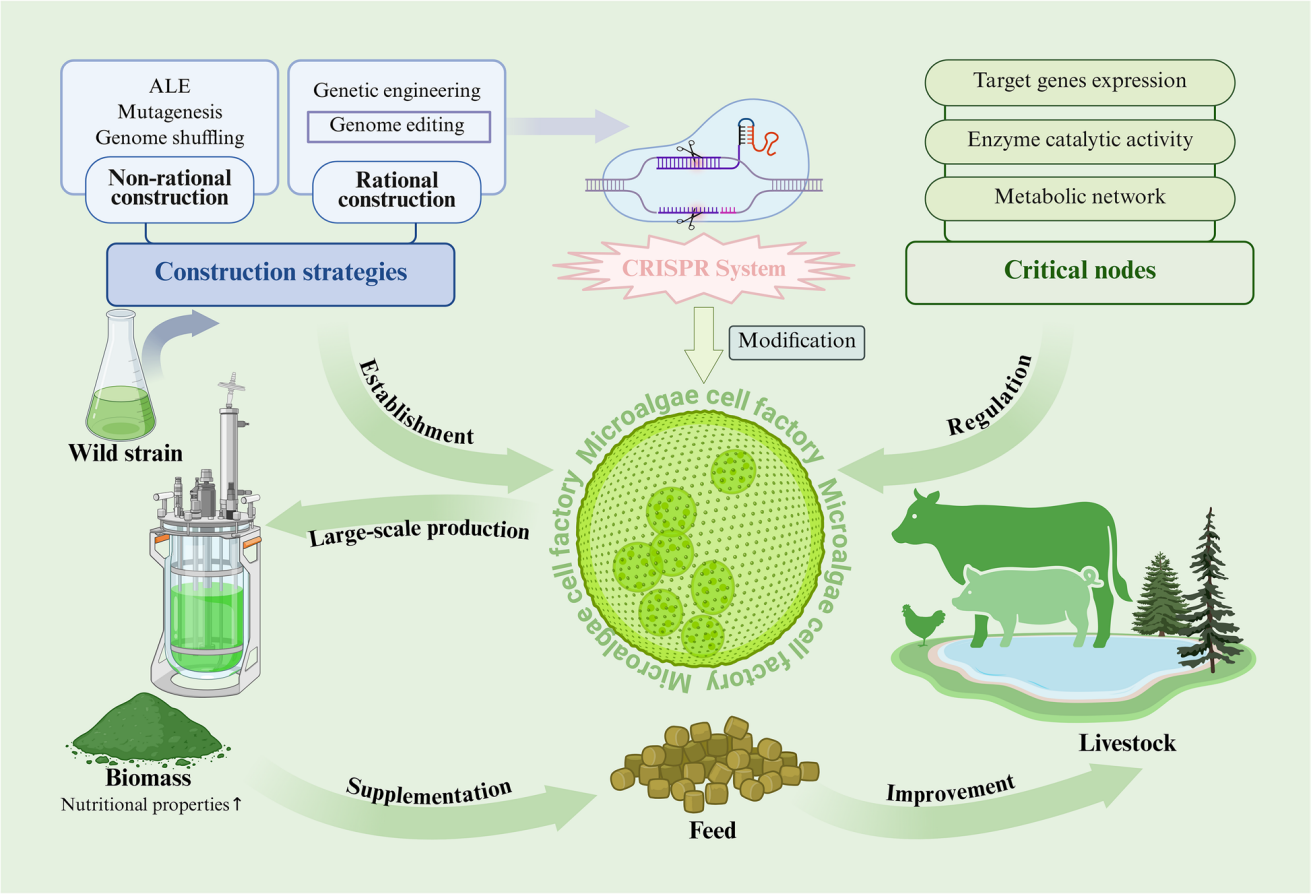

## Introduction

With the growing demand for sustainable biological resources, microalgae have been widely utilized in various fields such as biofuel production, high-value compound extraction, and environmental treatment (bioremediation of petroleum hydrocarbons) [1]. Especially, amidst rapid population growth and pressing challenges in food security, environmental sustainability, and energy crises, microalgae as a microbial resource with unique biological characteristics have garnered significant attention. Microalgae exhibit remarkable advantages, including high photosynthetic efficiency, rapid growth rates, and strong environmental adaptability [1]. They can be cultivated using non-traditional resources such as mudflats, saline-alkali lands, seawater, and even industrial wastewater, thereby reducing the reliance of conventional agriculture on arable land and freshwater resources. Notably, microalgae are rich in high-value bioactive compounds, such as high-quality protein (accounting for 50%–70% of dry weight) [1], polyunsaturated fatty acids (PUFAs) (e.g., docosahexaenoic acid [DHA] and eicosapentaenoic acid [EPA]) [2], carotenoids (e.g., β-carotene and astaxanthin) [3], and vitamins [2]. These components play an important role in enhancing livestock growth, boosting immunity, and improving product quality [4, 5].

The global feed industry (swine, poultry, ruminants and aquatic animals) is undergoing major transformations. Increasing consumer demand for healthy, safe, and sustainable animal products is driving a shift from synthetic to natural ingredient. Under the carbon neutrality strategy, developing sustainable feed resources has become an industry imperative. Microalgae cell factories present a promising solution to these challenges due to their unique advantages, such as coupling CO_2_ fixation with high-value compound production, as well as superior cost efficiency and functionality compared to traditional microbial systems [6, 7]. However, industrial-scale microalgae production still faces bottlenecks, including low productivity from poor performance of species, stringent cultivation requirements, and contamination risks, limiting their widespread adoption in the feed industry [8]. The key to overcoming the bottlenecks in commercial microalgae bioactive production lies in leveraging biotechnology to develop high-yielding microalgal cell factories. Advances in synthetic biology offer innovative solutions to these challenges. By systematically optimizing the metabolic networks of microalgal chassis cells, the yield and production efficiency of target compounds can be significantly enhanced [9]. Notably, emerging genome editing technologies like CRISPR enable precise synthetic pathway engineering and nutritional profile optimization in microalgae [10, 11]. These technological breakthroughs provide unprecedented opportunities for constructing efficient microalgal production platforms and developing next-generation functional feed ingredients.

Therefore, this article comprehensively reviews the current applications of microalgal cell factories, outlines key construction strategies, and systematically states critical nodes in microalgal biosynthetic pathways. Special focus is placed on CRISPR-based approaches for enhancing the nutritional profile of microalgae. Furthermore, we evaluate recent advances in microalgal applications for livestock production. By analyzing current technological limitations and knowledge gaps, we discuss the challenges and future directions for microalgae-derived bioactive compounds in animal nutrition, offering valuable insights to accelerate the industrialization of microalgae-based functional feeds.

## Biosynthesis based on microalgae cell factory

### Microalgae cell factory

The conventional chemical industry synthesizes ammonia and other compounds primarily through catalytic processes. In contrast, biological approaches utilizing enzymes or microbial systems offer distinct advantages, including mild reaction conditions, high substrate specificity, and lower energy use. Microbial cells inherently possess thousands of biochemical reactions and transport processes, with organelles acting as specialized production units, enabling whole cells to function as efficient bioreactors for sustainable biomanufacturing. Microalgae are unique photosynthetic cell factories capable of autotrophic growth, with each cell acting as an autonomous biosynthetic platform. Through systematic investigation of the biosynthetic elements, metabolic pathways, regulation and stress response in chassis microalgae, synthetic biology tools have been employed to design and construct novel, high-performance cell factories.

### Application of microalgae cell factory in biosynthesis

**Fig. 1:**
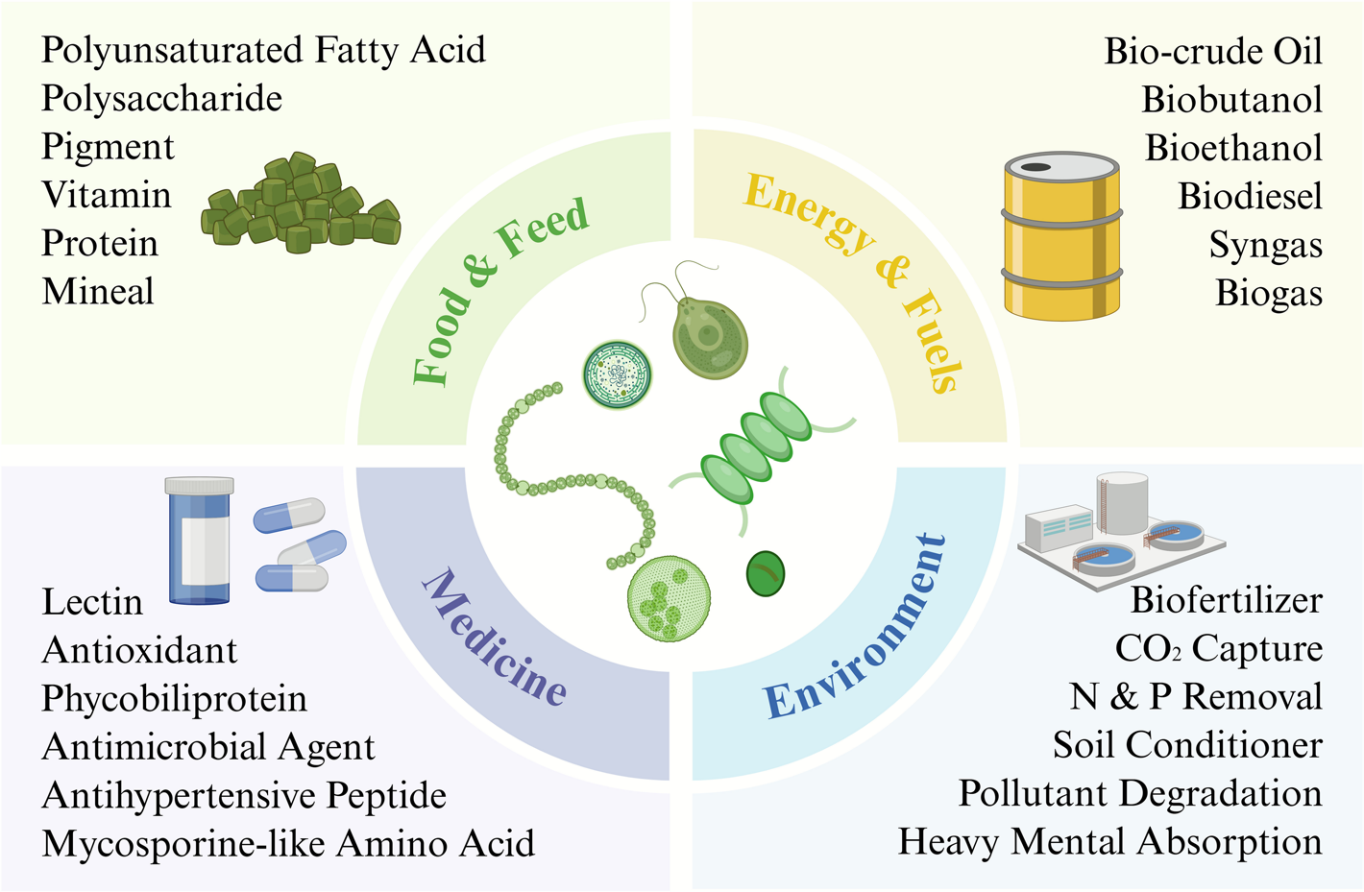
The versatile applications of microalgae cell factory

Through their inherent biosynthetic and metabolic pathways, microalgae-based cell factories demonstrate significant potential for diverse applications (Fig. 1), including the high-value bioactive compound synthesis, environmental bioremediation, and sustainable biofuel generation.

**Fig. 2:**
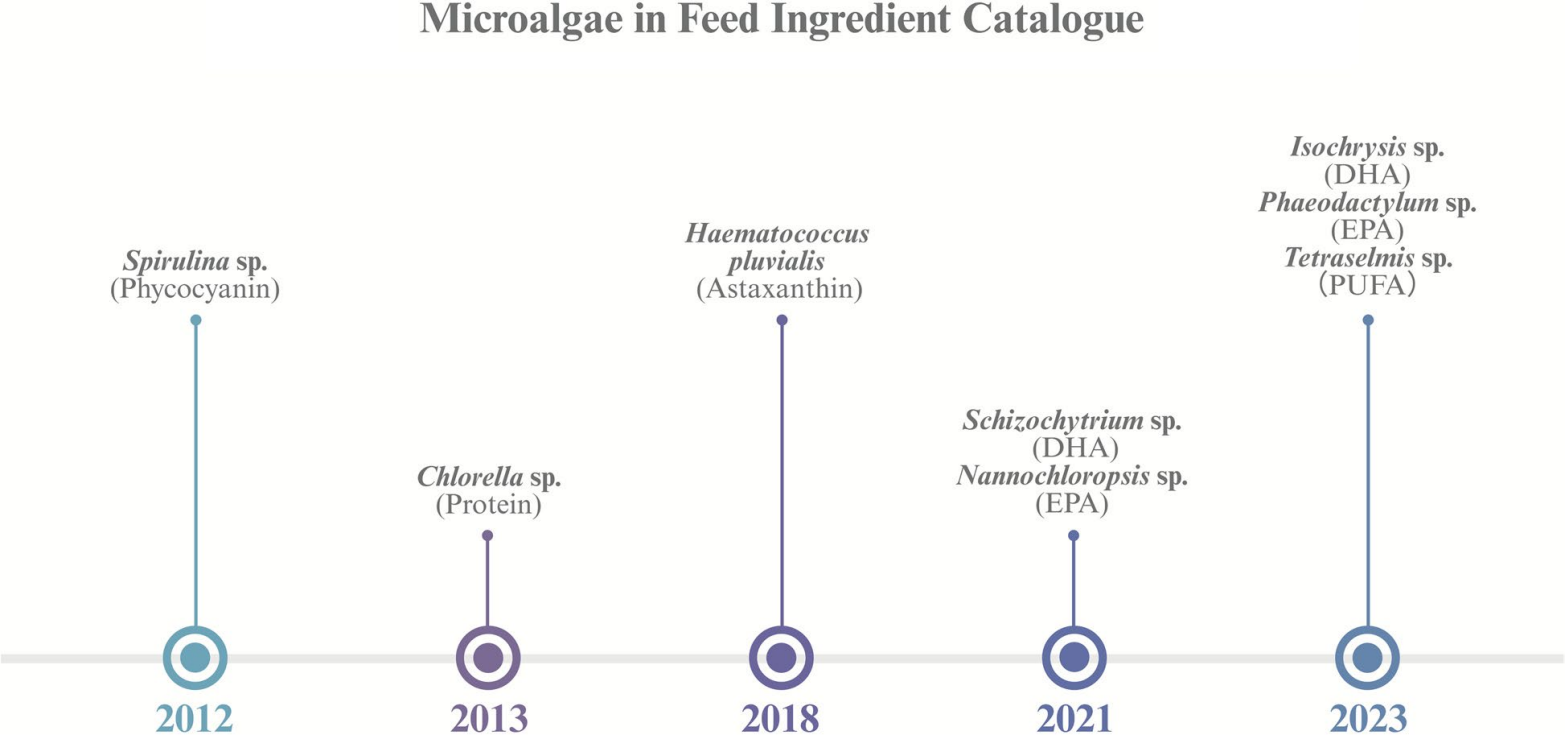
Microalgae species utilized as feed in China

Compared to conventional cell factories like *Escherichia coli* and *Saccharomyces cerevisiae*, microalgae exhibit slower growth rates and lower product concentration, making it necessary to develop specific high-value products suited to them. Notably, protein-rich species including *Spirulina platensis* (approved in 2012) and *Chlorella pyrenoidosa* (approved in 2013) are listed in China's feed materials catalogue. *Dunaliella salina*-produced β-carotene and *Haematococcus pluvialis*-derived astaxanthin are extensively utilized as nutritional additives in food and feed. Since the 1990s, research has established the critical health benefits of microalgal PUFAs, particularly DHA and EPA that are widely used in fish nutrition [12]. Additionally, advances in microalgal biotechnology have characterized multiple bioactive metabolites (e.g., immunomodulatory polysaccharides, antioxidant pigments, and essential PUFAs) with demonstrated utility as drug discovery targets and active cosmetic ingredients. Currently, approved species in the catalogue include *Schizochytrium* sp., *Nannochloropsis gaditana*, *Phaeodactylum* sp., *Isochrysis* sp., and *Tetraselmis* sp. (Fig. 2). The contents of select bioactive products are summarized in Table 1.

**Table 1:** The content of biosynthesis of bioactive compounds by microalgae

Microalgae species	Biomass, g/L	Bioactive product	Content, mg/g	Reference
*Chlorella protothecoides* CS-41	21.20	Lutein	138.15	[17]
*Chlorella sorokiniana* Kh12	4.61	Lutein	17.30	[18]
*Chlorella sorokiniana* FZU60	4.27	Lutein	11.22	[19]
*Haematococcus pluvialis*	1.43	Astaxanthin	29.91	[20]
*Chlorella zofingiensis*	3.3	Astaxanthin	16.70	[21]
*Chlamydomonas reinhardtii*	NA	β-Carotene	30.65	[22]
*Dunaliella salina*	NA	β-Carotene	25.23	[23]
*Amphidinium carterae*	NA	Peridinin	32.44	[24]
*Nannochloropsis oceanica*	4.5	Canthaxanthin & EPA	37.6 & 268.8	[25]
*Tisochrysis lutea & Microchloropsis salina*	4.2	DHA & EPA	26 & 23	[26]
*Chlorella saccharophila*	NA	Zeaxanthin	11.2	[27]
*Nannochloropsis oceanica* WS-1	0.21	Violaxanthin	5.21	[28]
*Isochrysis galbana*	2.13	Fucoxanthin	21.02	[29]
*Tisochrysis lutea*	3.17	Fucoxanthin & DHA	4.24 & 30.64	[30]

Anthropogenic global climate change is a major scientific and societal concern. Microalgae, with their rapid growth rates and ability to utilize nitrogen and phosphorus from wastewater while sequestering CO_2_, offer promising environmental remediation [13]. They can be effectively incorporated into circular bioeconomy systems, transforming wastewater and flue gases into valuable biomass [14]. Coupling microalgae cultivation with solar technologies reduces the carbon footprint and supports a low-carbon circular economy. A green process route was proposed by Liu et al. [13] that *Chlorella* sp. was used to immobilize CO_2_ from ethanol fermentation, ultimately producing ethanol products and biomass. Similarly, Xin et al. [14] enhanced *Euglena gracilis* CO₂ fixation efficiency by 147% using nuclear mutagenesis and adaptive laboratory evolution (ALE) approach. Since microalgal CO₂ capture capacity scales with growth rate, optimizing biomass productivity is key to enhance sequestration efficiency.

Fossil fuels particularly oil and coal comprise over 50% of China’s primary energy consumption, despite global commitments to phase out coal under the Glasgow Climate Pact [15]. Therefore, it is urgent to seek alternative energy sources and establish a new energy structure. Microalgae cell factories are considered a promising biofuel feedstock. Shin et al. [16] achieved a 2.1-fold enhancement in lipid accumulation (reaching 41.29% of dry weight) in *C. reinhardtii* through overexpressing glyceraldehyde-3-phosphate dehydrogenase. Despite certain microalgal strains exhibiting high intrinsic lipid content, their low productivity and the need for long-term nitrogen deprivation or other stress conditions lead to high production costs for microalgae-based biodiesel.

Currently, microalgae industrial-scale production also faces significant challenges such as poor environmental stress tolerance, the requirement for two-stage cultivation (biomass accumulation followed by product induction) and complex operations. Moreover, only a few microalgae strains are suitable for food or feed. The key to scaling lies in using biotechnology to construct microalgae cell factories with rapid growth, robust environmental resilience, and high target product accumulation.

## Construction strategies of microalgae cell factory

Developing high-performance strains is crucial for the application of microalgae biotechnology. Enhancing the content and yield of high-value metabolites in microalgae significantly improves industrial profitability, while reducing chlorophyll levels lowers downstream processing costs like decolorization. The development of commercially viable algal strains is a prerequisite for successful microalgae industrialization. Common breeding approaches encompass various synthetic biology techniques, including ALE, mutagenesis, genome rearrangement, genetic engineering, and genome editing. Integrating these methods can significantly enhance the efficiency of microalgae cell factory development.

### Common chassis microalgae

Several microalgal species, including *Synechococcus* sp., *C. reinhardtii*, and *Microcystis aeruginosa*, have been extensively studied as chassis organisms. These microalgae were among the first to be sequenced, with numerous genetic transformation methods and gene editing tools developed. As shown in Table 2, the genomes of *Synechococcus* sp. are significantly smaller than those of eukaryotic microalgae, with relatively complete gene annotation. The construction of chassis cells typically involves three key strategies: (1) reinforcement of endogenous metabolic pathways, (2) introduction of heterologous genes, and (3) construction of novel synthetic pathways. For effective cell factories, it is essential to improve growth phenotype, enhance light-use efficiency, boost carbon fixation and stress resistance [31]. Notably, microalgae genomes contain substantial redundancy, and large genomes can complicate genetic engineering. Supporting this, Wang et al. [32] used CRISPR/Cas9 to knock out a 214 kb fragment of *Nannochloropsis oceanica* without impairing growth, indicating that removing non-essential genomic regions could create minimal, optimized chassis for next-generation cell factories.

**Table 2:** Common microalgae chassis genomic information

Microalgae species	Genome size, Mb	GC content, %	GenBank ID
*Synechococcus elongatus* PCC 7942	2.70	55.5	GCF_000012525.1
*Synechocystis* sp. PCC 6803	3.90	47.5	GCF_000009725.1
*Nannochloropsis oceanica*	29.10	54.0	GCA_004519485.1
*Nannochloropsis gaditana* CCMP526	34.00	54.5	GCF_000240725.1
*Chlorella vulgaris*	40.20	62.0	GCA_023343905.1
*Chlorella variabilis*	46.20	67.0	GCF_000147415.1
*Chlorella sorokiniana*	61.50	66.5	GCA_034642255.1
*Chlamydomonas reinhardtii*	111.10	64.0	GCF_000002595.2
*Haematococcus lacustris*	309.40	60.0	GCA_030144725.1
*Phaeodactylum tricornutum* CCAP 1055/1	27.50	49.0	GCA_000150955.2

### Non-rational construction of microalgae cell factory

#### Adaptive laboratory evolution

ALE is an established method for developing industrially relevant metabolic phenotypes and enhancing microbial tolerance to non-native carbon sources, environmental stresses, and chemical inhibitors [33]. It directs microbial evolution over successive generations. Unlike genetic engineering, ALE circumvents limitations of host genetic background and complex phenotype interactions. While traditionally applied to *S. cerevisiae* and *E. coli* improvement, ALE has recently been successfully implemented in microalgae to optimize growth characteristics, enhance tolerance to flue gas or phenol and regulate lipid synthesis (Table 3).

**Table 3:** Advance in the research on ALE of microalgae

Microalgae species	Evolution conditions	Time, d	Target	Reference
*Cyanidioschyzon merolae*	10 mmol/L Ni	150	Enhance algal cell growth and reduce oxidative damage	[34]
*Synechococcus elongatu* PCC 7942	20% CO_2_	49	Increase biomass and lipid content	[35]
*Dunaliella salina*	500 mg/L phenol	210	Improve the phenol tolerance and degradation capability	[36]
*Chlorella* sp. AE10	10% CO_2_, 0.02% NO_x_ and 0.01% SO_x_	138	Improve the tolerance of flue gas	[37]
*Chlorella* sp. AE10	30 g/L NaCl and 10% CO_2_	138	Improve high salinity tolerance and high concentration CO_2_	[38]
*Nitzschia inconspicua*	37.5 °C	300	Improve the tolerance of temperature	[39]
*Schizochytrium* sp. HX-308	30 g/L NaCl	150	Improve cell growth and lipid production	[40]
*Crypthecodinium cohnii* ATCC 30556	Fermentation supernatant	840	Improve cell growth and DHA production	[41]
*Phaeodactylum tricornutum*	30 g/L glucose and temperatures (10 or 20 °C)	240	Improve cell growth and PUFA production	[42]
*Isochrysis galbana*	100 mg/L phenol	90	Improve phenol removal capacity	[43]

The success of ALE critically depends on suitable evolutionary conditions, categorized as stress-induced or non-stress-induced. Under stress, microalgae adapt their metabolism, often leading to the valuable compound production or pollutant degradation capabilities. Evolved strains typically maintain stable growth under stress and may even exhibit enhanced growth rates and higher yields of target metabolites under normal conditions [35]. Consequently, ALE is widely used to develop strains with faster growth, enhanced stress tolerance, higher production of high-value compounds and better pollutant degradation [44]. While most studies focus on stress-induced ALE, non-stress evolution (e.g., optimizing gene expression without growth inhibition) is also effective. A study showed that evolving *N. oceanica* under low salinity significantly improved its lipid accumulation [45].

ALE offers two key advantages for targeted strain improvement without requiring comprehensive prior knowledge of metabolic pathways and the facilitation of rapid genotype analysis through whole-genome sequencing. This approach effectively reveals evolutionary trajectories, identifies molecular mechanisms underlying strain improvement, and uncovers novel metabolic potential. However, its disadvantages are also obvious, including extended experimental duration (usually ≥ 30 generations for stable phenotypes), the need to purify target algal strains from mixed populations, and result unpredictability requiring repeated optimization [36].

#### Mutagenesis

Mutation breeding involves exposing cells to physical or chemical mutagens to induce genetic mutations, followed by screening for desired metabolic phenotypes [46]. This method features operational simplicity and can rapidly generate diverse mutant libraries for phenotypic selection. Common mutagens include chemical agents (e.g., nitrosoguanidine and ethyl methanesulfonate [EMS]) and physical approaches (ultraviolet [UV], ionizing radiation, laser, and atmospheric and room-temperature plasma [ARTP]). UV mutagenesis increased oil content in *Scenedesmus* sp. from 40% to 55% [46], while EMS boosted lutein and zeaxanthin productivity in *Dunaliella* sp. ZP-1 by over 20% [47]. As an emerging mutagenesis technology, ARTP offers distinct advantages, including rapid processing, low cost, and environmental friendliness. Notably, ARTP induces more severe genetic damage and higher mutagenic efficiency. The mutant strain of *C. pyrenoidosa* selected by Gong et al. [48] using ARTP had excellent growth performance and improved sensory and nutritional qualities, with a 16% increase in lutein content. For optimal mutagenesis outcomes, a post-mutagenesis survival rate of 5%–20% is recommended, followed by dark incubation to limit DNA repair.

Mutation generates extensive mutant libraries, making efficient screening crucial for identifying desired phenotypes. Phenotypic screening can focus on colony morphology, pigmentation alterations and fluorescence characteristics [50]. Environmental stress screening represents another effective secondary selection strategy, where mutants are subjected to controlled stress conditions to identify variants with enhanced tolerance [51]. Similar to ALE, mutation breeding is suitable for increasing microalgae biomass yield, promoting the production of specific biomolecules, and improving environmental tolerance without requiring prior genetic knowledge. Multiple rounds of mutagenesis are typically required to obtain desired mutant phenotypes [50]. To date, current mutagenesis has successfully generated algal strains with enhanced production of zeaxanthin, lutein, and astaxanthin (Table 4).

**Table 4:** Advance in the research on mutagenesis breeding of microalgae

Wild strain	Mutagenesis methods	Screening conditions	Target	Reference
*Graesiella emersonii*	ARTP	Larger diameters and darker pigmentation colonies on methanol plates	Improve protein content with superior amino acid profile and enhance astaxanthin content	[49]
*Auxenochlorella pyrenoidosa*	ARTP	First-round: certain yellow mutant, second-round: bigger diameter clones	A chlorophyll-deficient mutant	[50]
*Ulothrix* SDJZ-17	ARTP	Tolerance to low pH	Increase CO_2_ tolerance, photosynthetic efficiency and lipid productivity	[51]
*Dunaliella* sp. ZP-1	EMS	Based on colony color	Higher lutein and zeaxanthin productivity	[47]
*Chromochloris zofingiensis*	EMS	Golden yellow colonies	Increase lutein, zeaxanthin, and β-carotene content	[52]
*Chromochloris zofingiensis*	MNNG	The stay-green mutant	Increase lutein content	[53]
*Euglena gracilis*	^60^Co-γ	Highest growth rate and good polyethylene glycol adaptation	Promote cell proliferation and reduce the apoptosis and necrosis rates	[54]
*Tetraselmis suecica*	Ultraviolet	Higher Nile red fluorescence colonies	Improve lipid productivity	[55]

*ARTP* Atmospheric and room-temperature plasma, *EMS* Ethyl methanesulfonate, *MNNG* N-methyl-N’-nitro-N-nitrosoguanidine, ^*60*^*Co-γ* Cobalt-60 gamma-ray irradiation

#### Genome shuffling

Genome shuffling integrates multiple genetic traits into algal strains for phenotypic improvement, involving parental selection, protoplast preparation, fusion, and recombinant screening. Effective genome shuffling requires genotypically diverse parental strains, typically generated through prior mutagenesis. Empirical evidences suggest that combinatorial mutagenesis is more effective to accumulate abundant mutations. Through a three-phase mutagenesis protocol, Wang et al. [56] successfully enhanced astaxanthin production in *H. pluvialis*, ultimately achieving with a 1.7-fold increase relative to the wild-type strain.

Following parental strain selection, protoplast preparation necessitates complete cell wall removal, often achieved through enzymatic digestion tailored to the species. Commonly employed enzymes include cellulase, snail enzyme, pectinase, hemicellulase, lysozyme, and driselase. Xie et al. [57] effectively generated protoplasts from *Chlorella sorokiniana* using a combination of 4% cellulase and 2% driselase. The most prevalent protoplast fusion methods involve either chemical treatment or electrofusion. Polyethylene glycol with Ca^2+^ has been shown to significantly enhance protoplast fusion efficiency [58]. In addition, femtosecond lasers, optical tweezers, and microfluidic chips have also demonstrated promising results [59].

As a simple and cost-effective breeding strategy, genome shuffling can be effectively combined with other methods to engineer and optimize microalgal cell factories. In a systematic comparison, Fields et al. [60] found that combining genetic engineering, UV, and genome shuffling achieved the highest protein yields in *C. reinhardtii*.

### Rational construction of microalgae cell factory

#### Genetic engineering

Microalgae genome sequencing provides the essential molecular data foundation for targeted genetic engineering. The concurrent development of advanced genetic manipulation tools has enabled the systematic design of microalgal cell factories. While genetic engineering techniques for prokaryotic microalgae are mature, significant progress has been made with eukaryotic species (particularly *C. reinhardtii*, *M. aeruginosa*, and *Phaeodactylum tricornutum*), especially through genome editing. Whereas classical techniques (ALE, EMS mutagenesis, genome shuffling) create phenotypic diversity through random mutagenesis, synthetic biology tools (Golden Gate assembly, CRISPR/Cas9) enable precise genetic modifications at predetermined genomic loci.

**Fig. 3:**
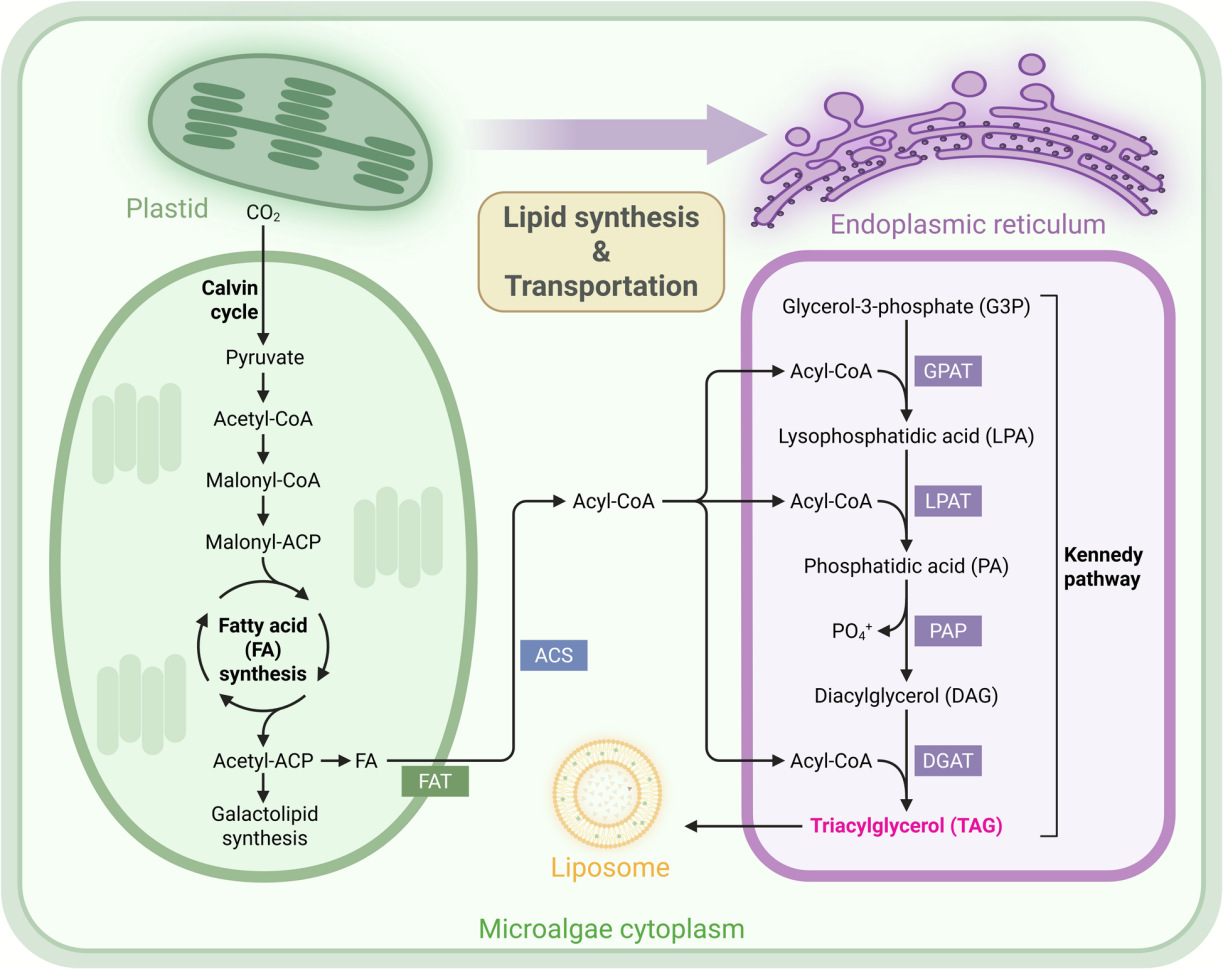
Microalgae lipid synthesis pathway

Genetic engineering involves introducing exogenous DNA into cells to enable its expression, primarily through nuclear transformation, chloroplast transformation, and RNA interference (RNAi). Nuclear transformation is the most widely used, employing various DNA delivery methods including glass bead agitation, electroporation, and particle bombardment. Jiang et al. [61] demonstrated this through electroporation-mediated plasmid transfer in the *P. tricornutum*, where expression of lipid-associated proteins enhanced fucoxanthin production by 51% compared to wild-type strains. The chloroplast transformation system in *C. reinhardtii* facilitates robust foreign gene expression without problematic glycosylation. This system leverages the plastid genome to achieve both proper post-translational processing and high protein yields. RNAi technology utilizes short (20–30 nt) regulatory RNA to silence genes via translational blocking, mRNA cleavage, or chromatin modification, though its use in microalgae remains limited to few model organisms. Overexpression strategies are also effective. Targeted overexpression of glycerol-3-phosphate acyltransferase of Kennedy pathway (Fig. 3) in *N. oceanica* significantly enhanced non-polar lipid accumulation under optimized culture conditions [62]. Similarly, overexpression of shikimic acid kinase in *Synechococcus elongatus* PCC 7942 by Sun et al. [63] enhanced the tolerance to high light and temperature more efficiently than traditional ALE.

#### Genome editing

Compared to traditional low-efficiency genetic engineering for microalgal cell factory development, genome editing provides a transformative alternative [64]. Current tools include homing endonucleases, transcription activator like effector nucleases (TALENs), zinc finger nucleases (ZFNs), and CRISPR/Cas9. However, TALENs and ZFNs face significant limitations by low editing efficiency and costly vector construction [11]. In contrast, the CRISPR/Cas9 system offers superior efficiency, specificity, and cost-effectiveness compared to protein-dependent editing tools [64]. This revolutionary system utilizes programmable guide RNAs (gRNAs) for target recognition rather than engineered proteins, enabling faster, more economical, and highly precise genome editing in microalgae. Song et al. [65] utilized CRISPR/Cas9 to knockout two genes in *C. reinhardtii* under nitrogen limitation, producing algal oil containing 8.42 mg/g lutein and 7.69 mg/g zeaxanthin. Recent advancements have further expanded the CRISPR toolkit, including CRISPRa/i, employing catalytically inactive Cas9 for gene regulation. Lin and Ng [66] applied CRISPRi to suppress phosphoenolpyruvate carboxylase in *C. reinhardtii*, redirecting carbon flux to achieve 34.88% lipid content and 9.18 mg/g lutein at 35°C.

## Critical nodes in synthesis pathways

**Fig. 4:**
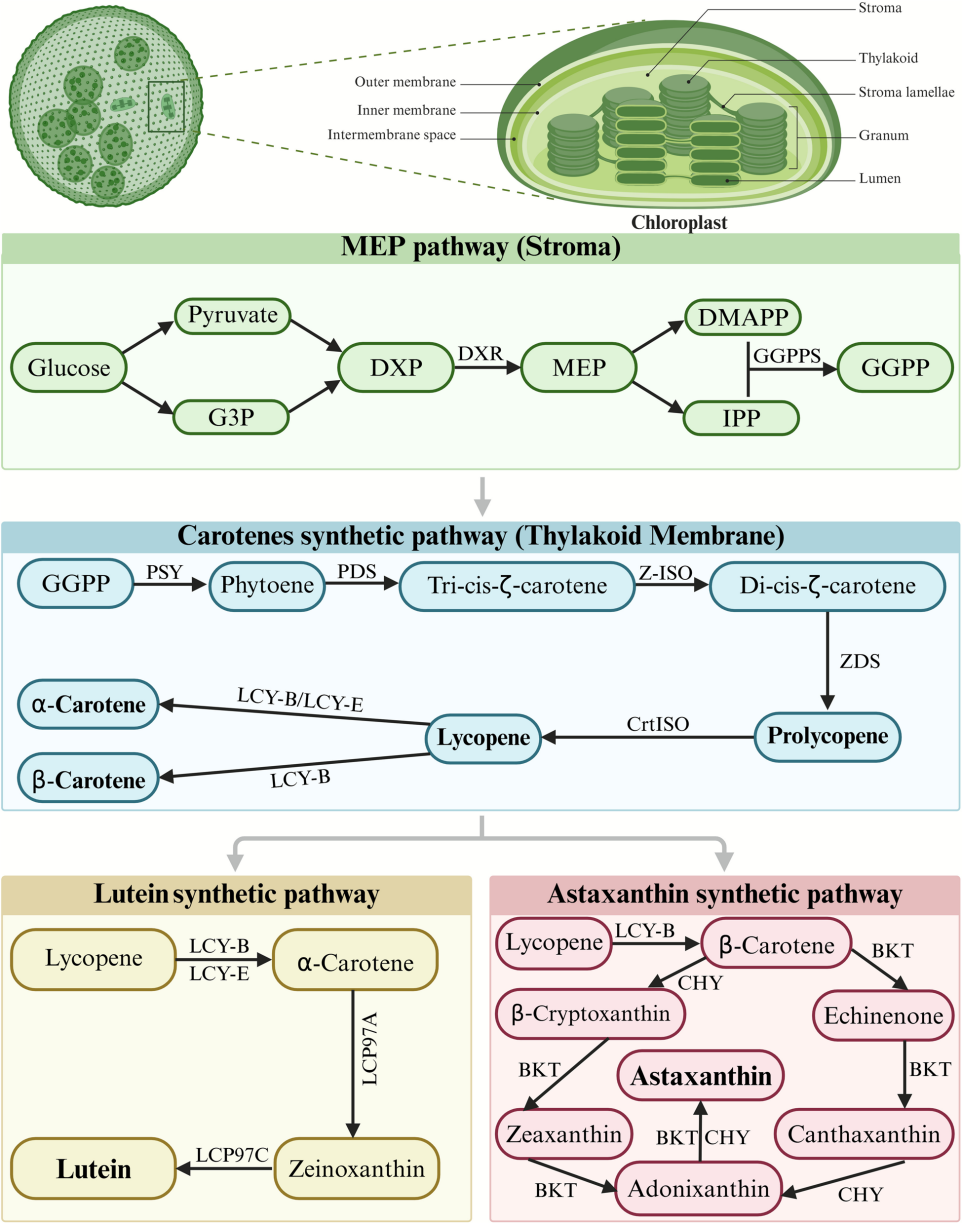
Biosynthesis pathways of carotenoid in microalgae. G3P, Glyceraldehyde 3-phosphate; DXP, 1-Deoxy-D-xylulose 5-phosphate; DXR, 1-Deoxy-D-xylulose 5-phosphate reductoisomerase; MEP, 2-C-Methyl-D-erythritol 4-phosphate; DMAPP, Dimethylallyl pyrophosphate; GGPPS, Geranylgeranyl pyrophosphate synthase; IPP, Isopentenyl pyrophosphate; GGPP, Geranylgeranyl pyrophosphate; PSY, Phytoene synthase; PDS, Phytoene desaturase; Z-ISO, ζ-Carotene isomerase; ZDS, ζ-Carotene desaturase; CrtISO, Carotenoid isomerase; LCY-B, Lycopene β-cyclase; LCY-E, Lycopene ε-cyclase; LCP97A, Cytochrome P450 family 97 subfamily A; LCP97C, Cytochrome P450 family 97 subfamily C; CHY, β-Carotene hydroxylase; BKT, β-Carotene ketolase

The construction of microalgae cell factories depends critically on target gene levels and key enzymatic activities. Additionally, excessive metabolite accumulation may disrupt cellular metabolic network, thereby impeding target product biosynthesis. Consequently, dynamic regulation of key biosynthetic nodes, especially in carotenoid (Fig. 4), represents an effective strategy for enhancing the production of high-value compounds in microalgae.

### Target gene expression

Enhancing gene regulation in microalgae involves strategies including promoter/terminator selection, intron mediation, and codon optimization. Pcpc560, a strong promoter derived from *Synechocystis* sp. PCC 6803, effectively drives exogenous protein expression in cyanobacteria [67] and chlorophyll production [68]. Engineered promoters also offer a promising approach to minimize transgenic silencing and boost gene expression. In *C. reinhardtii*, the synthetic AR1 promoter currently demonstrates the highest efficient, outperforming endogenous promoters such as the chimeric hsp70/rbs2 promoter [69]. Furthermore, synthetic promoters incorporating *cis*-regulatory elements exhibit greater potential for heterologous gene expression [70]. Introns play a crucial regulatory role in gene expression by improving transcription, nuclear export, mRNA stabilization, and translation efficiency [71]. Thus, the strategic insertion of optimized intron sequences into expression cassettes can significantly enhance transgene expression. In eukaryotic microalgae, intron-mediated enhancement has been successfully applied to boost heterologous gene expression, with demonstrated efficacy in *Scenedesmus acutus* [72], *C. reinhardtii* [73], and *F. solaris* [74]. Notably, the insertion position of introns also influences expression levels [73]. Additionally, codon optimization based on host-specific preferences can substantially improve transgene expression. Jaeger et al. [75] developed an online tool for designing codon-optimized sequences with integrated introns to enhance transgene expression in microalgae. Building upon this tool, Aschern et al. [76] developed an advanced strategy for intron insertion into synthetic DNA fragments to enhance NanoLuc expression with intron numbers increased.

During the construction of heterologous biosynthetic pathways in microalgae, transgene expression is frequently compromised by sRNA-mediated gene silencing. Silencing can result from factors like promoter selection, intron incorporation, sequence composition, terminator elements, and transcriptional termination mechanisms. A study has demonstrated that terminators help regulate siRNA accumulation and maintain transgene stability [77]. High GC content has been shown to enhance transgenic resistance to silencing while improving intergenerational stability [78]. Matrix attachment regions (MARs), defined as genomic DNA elements that bind to the nuclear matrix in chromatin non-coding regions, offer another effective strategy to counteract silencing effects [79]. MAR elements can counteract low-copy-number-induced homologous repression, boost transcriptional activity, and significantly improve both expression levels and stability of transgenes [80]. In *D. salina*, MAR incorporation resulted in a 4.5-fold increase in reporter gene expression coupled with a threefold reduction in transformant variability [81].

### Enzyme catalytic activity

Beyond transcriptional control, post-translational modulation of enzymatic activity constitutes a critical strategy for enhancing high-value compound production in microalgae. Multiple regulatory proteins fine-tune biosynthetic pathways in microalgae. The orange protein serves as a key regulator of phytoene synthase (PSY), stabilizing the enzyme complex and promoting carotenoid biosynthesis (Fig. 4) [82]. Overexpression of natural oranges and their mutants in *C. reinhardtii* increased carotenoid production to varying degrees [83]. In tetrapyrrole biosynthesis, the glutamyl-tRNA reductase (GluTR)-binding protein (GBP) mediates feedback inhibition of GluTR through competitive heme binding, which triggers GluTR degradation [84]. This suggests that modulating GBP expression could alleviate heme-mediated repression of porphyrin synthesis. The FLU regulatory protein provides heme-independent control by directly inactivating GluTR [85], forming a dual regulatory system to coordinate 5-aminolevulinic acid synthesis [86]. At the magnesium branch point, genome uncoupling 4 regulates chlorophyll synthesis by stabilizing Mg-chelatase complexes in both *C. reinhardtii* [87] and *Synechocystis* sp. PCC 6803 [88], while simultaneously protecting the CHLH-PPIX complex from photooxidation [87]. Additional regulation occurs through light-harvesting-like protein 3-mediated control of geranylgeranyl reductase and protochlorophyllide oxidoreductase activities, and through companion proteins that modulate tetrapyrrole flux [89, 90]. Furthermore, post-translational modifications, particularly thioredoxin-mediated redox regulation and phosphorylation, critically influence pathway enzymes. The coordinated action of two distinct thioredoxin systems, the iron-dependent ferredoxin-dependent thioredoxins (TRX) and NADPH-dependent TRX reductase C (NTRC) pathways, maintains proper chlorophyll biosynthesis, with knockout studies demonstrating their essential role in sustaining metabolic flux [91, 92].

Overexpression of enzymes in the synthesis pathway in microalgae cells may induce feedback inhibition, thereby limiting the production of target bioactive compounds. This can be addressed by expressing feedback-resistant enzyme variants or disrupting transcriptional and allosteric regulation. Expression of feedback-resistant enzyme variants in *Synechocystis* sp. PCC 6803 has been shown to significantly enhance target compound yields, with reported increases of 2–5-fold compared to wild-type systems [93, 94]. Disrupting enzyme‑inhibitor binding sites is another effective way to reduce feedback inhibition, supporting the development of efficient cell factories for metabolite overproduction. Furthermore, the de novo design of enzymes can enhance catalytic activity and eliminate inhibition. A notable demonstration of this approach involved expression of the artificially designed synthetic PSY variant in *Scenedesmus obliquus*, which achieved a carotenoid yield of 31.8 mg/g dry cell weight with a threefold increase over wild-type levels [95].

### Metabolic network

Metabolic pathway modifications often fail to achieve expected yields due to challenges including low translation efficiency of pathway genes, toxic intermediate cumulation, enzyme inhibition, substrate competition, and pathway imbalance [96]. To address these limitations, precise balancing metabolic fluxes is critical for constructing industrially viable microalgal cell factories. Inducible promoters offer a promising solution by enabling controlled gene expression under specific conditions, thereby reducing cellular toxicity and enhancing product yields [67]. For instance, a library of diverse inducible promoters has been established in *Synechocystis* sp. PCC 6803 [67], while the Fe^3+^-inducible Fea1 promoter has been functionally validated in *C. reinhardtii* for regulating nuclear transgenes [97]. Additionally, transcription factors (TFs) can dynamically modulate gene expression based on biochemical cues. Coupling TFs with promoters enables precise co-regulation of transgenes. In *E. coli*, a library of ligand-responsive allosteric TFs was developed to programmatically tune gene expression levels through engineered promoter systems [98, 99].

Riboswitches modulate gene expression at the translational level through ligand binding. In *C. reinhardtii*, the thiamine pyrophosphate (TPP) riboswitch that was first identified there [100] have since been modified to control terpenoid biosynthesis [101]. These riboswitches exhibit dose-dependent responses to ligands, enabling precise regulation of metabolic enzyme expression. Similarly, synthetic theophylline-responsive riboswitches have proven effective in cyanobacteria [102]. In *Synechocystis* sp. PCC 6803, a theophylline-induced riboswitch was used to control the Hfq-MicC system, thereby modulating carbon metabolic flux [103]. Furthermore, the engineered riboswitch theoE*, combined with T7 RNA polymerase in *S. elongates* UTEX 2973, formed an artificial induction system capable of dual-level (transcriptional and translational) regulation of transgenes [104]. Beyond riboswitches, biosensors represent an emerging tool for dynamic metabolic flux control. A dual-functional biosensor was developed in *E. coli*, leveraging the linearity between metabolic precursor and end-products, along with transcription factor-mediated promoter regulation, to achieve rapid and precise flux sensing and adjustment [105]. Similarly, a gene circuit based on *Bacillus cereus* alpha-1,2-fucosyltransferase enabled dynamic transcriptional and translational regulation of target genes in a pathway-independent manner, offering potential applications in microalgal target product metabolism [106]. Additionally, synthetic circuits based on quorum sensing have been integrated into metabolic engineering strategies to dynamically regulate multiple fluxes [107]. This approach has been successfully implemented in *E. coli* [108].

## Application of CRISPR system to optimize the feed characteristics of microalgae biomass

CRISPR currently stands as the most versatile and efficient genome editing platform, enabling precise modifications including gene knockout/knock-in, targeted mutagenesis, chromosomal rearrangement, and transcriptional regulation [109]. This technology has emerged as a transformative tool for engineering microalgal metabolic networks, with successful applications in modifying bioactive compound biosynthesis pathways to optimize microalgal feed characteristics (Table 5). The primary CRISPR systems employed for microalgal metabolic engineering including CRISPR/Cas9, CRISPR-Cas12a, CRISPRa/i, and Cas-ribonucleoprotein (RNP).

**Table 5:** Application of CRISPR system in the enhancement of feed characteristics in microalgae

Microalgae species	CRISPR system	Bioactive component	Target gene	Efficiency, mg/L	Reference
*Chlamydomonas reinhardtii*	CRISPR-Cas9	Zeaxanthin	*ZEP*, *LCYE*	21.68	[110]
*Chlamydomonas reinhardtii*	CRISPR-Cas9	Terpenoids	*LHCBM1*	4.5	[111]
*Chlamydomonas reinhardtii*	CRISPR-Cas9	Glycolate	*CrHPR1*	280.1	[112]
*Chlamydomonas reinhardtii*	CRISPR-Cas9	Astaxanthin	*CrBKT*, *PacrtB*, *CrCHYB*	1.8	[113]
*Chlamydomonas reinhardtii*	CRISPR-Cas9 RNP	Spermidine	*SPD1*	0.75	[114]
*Phaeodactylum tricornutum*	CRISPR-Cas9	Lipid	*PtECH*	NA	[115]
*Phaeodactylum tricornutum*	CRISPR-Cas9	Mannose type N-glycan	*PtFucT1*	NA	[116]
*Phaeodactylum tricornutum*	CRISPR-Cas9	Lipid	*Pt2015*	NA	[117]
*Phaeodactylum tricornutum*	CRISPR-Cas9	NA	*LHC*	NA	[118]
*Phaeodactylum tricornutum*	CRISPR-Cas9 RNP	Phycoerythrin	*CHS1*	37.03	[119]
*Synechococcus elongatus*	CRISPRi-dCas 12a	Phycobiliprotein	*acnB*, *cpcB2*	NA	[120]

CRISPR/Cas9 represents the most widely adopted and well-characterized system in CRISPR-based genome editing. The Cas9 endonuclease induces targeted DNA double-strand breaks guided by a single-guide RNA (sgRNA), with subsequent repair occurring primarily through non-homologous end joining (NHEJ) or homology-dependent recombination (HDR). In microalgal metabolic engineering, NHEJ-mediated CRISPR/Cas9 has been predominantly employed for the accumulation of bioactive substance [110]. Disruption of β-carotene hydroxylase (CHYB) via CRISPR/Cas9 effectively reduced the expression of CHYB, which in turn remarkably increased β-carotene production in *D. salina* [121]. A recent study found that knockout of CHYB by CRISPR/Cas9 partially reduced lutein content and achieved high-density cultivation of *C. reinhardtii* [122]. Targeted knockout of the carboxyltransferase interactor 1 gene by the CRISPR/Cas9 system increased lipid yields in mutant strains, accumulating 25% more total fatty acids and storing up to fivefold more triacylglycerols [123]. While HDR-mediated editing remains challenging in microalgae due to donor template instability and copy number variability, recent advances demonstrate its potential. The integration of CRISPR-Cas9/HDR with a twin-viral replicon system significantly boosted carotenoid production in plant callus, providing a strategic framework for adapting HDR in eukaryotic microalgae [124]. Notably, knockout of KU70/KU80—key NHEJ pathway components—can enhance HDR and CRISPR system efficiency [125]. Moreover, CRISPR/Cas9 application reduced the cell wall thickness and cellulose content of *Nannochloropsis salina* and enhanced the lipid extractability, possibly resulting in the improvement of digestibility in vivo [126].

Compared to CRISPR/Cas9, CRISPR-Cas12a offers distinct advantages, including tracrRNA independence, higher editing precision, and significantly reduced cellular toxicity. This system has enabled marker-free genome editing in cyanobacteria [127]. Recently, a Cas12a-based toolkit combining RNP delivery with homologous directed recombination was developed for microalgae, achieving successful gene editing in *N. salina* [128]. However, the relationship between cleavage-to-mutation distance and editing efficiency in CRISPR-Cas12a systems remains poorly characterized, highlighting an opportunity to optimize this platform’s performance. The genomic integration of Cas protein and sgRNA genes often reduces the viability of transgenic microalgal strains. To circumvent this limitation, an alternative approach involves in vitro transcription and pre-assembly of Cas protein with sgRNA to form RNP complexes, which are then delivered into algal cells via electroporation or biolistic transformation. Notably, a novel triple nanocomplex system integrating cell-penetrating peptides, nuclear localization signal peptides, and plasmid DNA (utilizing pVEC-ORI and pVEC-R6A) has been successfully implemented in *C. reinhardtii* [129]. CRISPR-RNP systems offer the key advantages of transient Cas9 activity that minimizes off-target effects [130] and successful applications in enhancing pigment production in microalgae including *C. reinhardtii* [131], *E. gracilis* [132] and *Porphyridium* sp*.* [119].

In contrast to conventional CRISPR systems that modify DNA sequences, the CRISPRa/i system comprises catalytically inactive dead Cas9 (dCas9) and gRNA to regulate transcription without genetic alteration [133]. Guided by gRNA, dCas9 specifically binds to the promoter or coding sequence of the target gene, blocking transcription initiation or elongation. Additionally, dCas9 can bind to transcription factors or transcriptional repressors of target genes, effectively regulating their expression. CRISPRa/i has been widely adopted for metabolic network optimization in microalgae, enabling breakthrough productivity of target compounds [133]. In *C. sorokiniana*, CRISPRa/i-mediated engineering significantly enhanced the production of proteins and lipids [133]. The application of CRISPRa-VP64 activation increased protein content to 60% of dry cell weight, achieving a concentration of 570 mg/L. More notably, CRISPRi-KRAB inhibition, demonstrated superior efficacy by further elevating protein content to 65% and concurrently stimulating lipid synthesis within the range of 150–250 mg/L [133]. These results highlight the potential of CRISPR tools in microalgae metabolic engineering, helping to develop high-value microalgae feed.

Traditional CRISPR-Cas systems are widely used in microalgae engineering but face key limitations: strict protospacer adjacent motif (PAM) requirements and off-target effects. Overcoming PAM constraints is crucial to enhance CRISPR’s efficiency in microalgae. Zhao et al. [134] created CAGO, a gRNA-free editor using a universal N20 sequence for one-step modification. Protein engineering of Cas9 has also broadened PAM compatibility—e.g., Walton et al. [135] developed SpG and SpRY variants with relaxed PAM specificity. Building on SpRY, researchers later established base editors for plants [136]. These advanced CRISPR platforms enable targeted modification of diverse metabolic pathways in microalgae, significantly broadening the range of producible bioactive compounds while enhancing both yield and product specificity to optimize the feed characteristics of microalgae. CRISPR-Cas9 RNP knockout of *AGP*, a key enzyme in starch synthesis, generated a starch-deficient mutant of *Tetraselmis* sp. which showed significantly increased lipid content—up to 3.1 times that of the wild type under nitrogen stress, demonstrating its potential for sustainable lipid production [137]. Despite the commercial utilization of microalgae like *Chlorella* sp., *S. platensis*, and *H. pluvialis* for high-value feed additives, species-specific CRISPR editing tools remain underdeveloped. The current lack of tailored CRISPR gene editing tools for most industrially relevant microalgae species poses a major bottleneck. Addressing this gap through development of optimized CRISPR systems is critical for efficient synthesis of bioactive substances by microalgae for large-scale application in animal nutrition.

## Advancements in microalgae applications for livestock husbandry

The scale of livestock breeding continues to expand annually, making feed—a critical pillar of the industry—essential for achieving sustainable development. To ensure a stable global food supply, priority should be given to allocating land and water resources to food production rather than feed production. Microalgae-based biosynthesis represents a sustainable non-food biomass solution, offering high nutritional density, biosafety, yield reliability, and environmental benefits that make it ideal for functional feed ingredients or additives. Table 6 summarizes recent advances in the application of microalgae in livestock production.

**Table 6:** Applications of microalgae in husbandry production

Category	Microalgae	Inclusion level	Animal	Impact	Reference
Swine	*Chlorella vulgaris*	5%	Xiangcun black weaned piglet	Reduced the diarrhea rate and improved immune function and intestinal health	[138]
	*Chlorella vulgaris*	5%	Yorkshire × Landrace weaned piglets	Increased immunoglobulin levels and benefited hepatic function and n-6/n-3 ratio	[139]
	*Spirulina* sp.	10%	Pietrain × (Yorkshire × Landrace) weaned piglets	Improved liver metabolism by enhancing fatty acid breakdown, sugar processing, and cellular protection	[140]
	*Spirulina* sp.	0.025%–0.1%	(Landrace × Yorkshire) × Duroc growing pigs	Enhanced growth performance, nutrient digestibility, antioxidant enzyme activity, and fecal *Lactobacillus* counts	[141]
	*Schizochytrium* sp.	7%	Landrace weaned piglets to growing-finishing pigs	Increased muscle DHA and EPA contents to optimize lipidome composition	[142]
	*Schizochytrium* sp.	0.5%–1.0%	(Landrace × Yorkshire) × Duroc weaned piglets	Increased nutrient digestibility and lymphocyte concentration	[143]
	*Haematococcus pluvialis*	0.025 g/kg	Polish Landrace weaned piglets	Enhanced the intestinal wellness and productivity	[144]
	*Laminaria digitata*	10%	Yorkshire × Duroc weaned piglets	Improved both cellular and humoral immune response and cardiovascular health	[145]
Poultry	*Limnospira platensis*	15%	Ross 308 broilers	Improved nutrient utilization and meat quality with higher yellowness, more chlorophyll and carotenoids, and better fatty acids	[146]
	*Spirulina platensis*	2.5–10 g/kg	White Leghorn laying hens	Improved the productivity, eggs’ quality, shelf life, and blood biochemistry	[147]
	*Arthrospira platensis*	0.25–1.00 g/kg	Ross 308 broilers	Enriched the breast musculature in n-3 and n-6 PUFA and boosted immune response	[148]
	*Spirulina platensis* and* Chlorella vulgaris*	1.5 g/kg + 1.5 g/kg	Arbor Acres broiler	Improved antioxidant status in breast and thigh meat, intestinal morphology and intestinal flora	[149]
	*Spirulina platensis* and *Dunaliella salina*	0.5–2.0 g/kg	Japanese quails	Enhanced body weight, improved lipid metabolism, strengthened immune response, and optimized hepatic/renal functions	[150]
	*Parachlorella* sp.	0.5%	Ross 308 broilers	Promoted gut microbial colonization and intestinal immunity development	[151]
	*Nannochloropsis oculata*	0.5%–1.0%	Browne Lohmann laying hens	Improved the productivity, egg quality and antioxidant status	[152]
	*Schizochytrium*	20 g/kg	Ross 308 broilers	Improved the final weight, antioxidant ability and meat fatty acid composition	[153]
	*Haematococcus pluvialis*	25–100 mg/kg	Jinghong No.1 laying hens	Improved semen quality by increasing antioxidant ability	[154]
	*Haematococcus pluvialis*	20–160 mg/kg	Jinghong No.3 laying hens	Improved free radical scavenging ability and antioxidant enzyme activity	[155]
Ruminant	*Schizochytrium* sp.	2%	Tan sheep	Optimized the lipid profile of meat to improve meat quality	[156]
	*Schizochytrium* sp.	1.65%	Angus × Simmental heifers	Improved body weight and progeny birth weights	[157]
	*Schizochytrium* sp.	2%	Holstein calves	Decreased the serum lipopolysaccharide content and alleviated calf diarrhea	[158]
	*Schizochytrium* sp.	20–60 g/d	Alpine × Local (Greek) dairy goats	Reduced the relative abundances of *Methanobacterium formicicum* and Archaea to modify the rumen microbiome	[159]
	*Spirulina platensis*	20%–80%	Najdi growing lambs	Increased gain weight, white blood cells, specific immune factors and the abundance of beneficial bacteria	[160]
	*Spirulina* sp.	3%	Hu sheep	Improved rumen development and fermentation, and effectively relieved rumen microbe disorders	[161]
	*Spirulina* sp.	5–15 g/d	Chios ewes	Improved the ewes’ oxidative status of their organism and milk	[162]
	*Spirulina* sp.	1%–3%	Hu sheep	Ameliorated lipid metabolic disorder and oxidative stress	[163]
	*Spirulina platensis* and* Chlorella vulgaris*	0.1 g/kg	Ossimi sheep	Boosted immunity, improved growth, and enhanced overall health by reducing heat and oxidative stress damage	[164]
	*Chlorella vulgaris*	0.5%–1.0%	Merino × Dorper lambs	Elevated α-linolenic acid and total n-3 PUFA in fresh meat, with no impact on performance or carcass traits	[165]

Traditional livestock farming primarily relies on grains and legumes as feed ingredients [166]. However, microalgae can serve as a sustainable protein source to partially replace conventional feed [166]. Rich in vitamins, minerals, and essential amino acids such as methionine and lysine—which are often deficient in standard diets—microalgae can enhance meat and product quality when used as a supplement. Additionally, it reduces dependence on costly synthetic nutritional additives in animal feed formulations [4]. Soybean meal partially replaced by *C. vulgaris* reduced the diarrhea rate of weaned piglets without significantly affecting on growth performance [138]. A study showed that supplementing laying hen diets with *S. platensis* could partially replace soybean meal while enhancing production performance and egg quality [167]. However, a report on broilers indicated that incorporating *Arthrospira* spp. as a partial substitute for soybean-based feed during the first 22 d post-hatching significantly impaired growth performance, regardless of inclusion level [168]. These findings suggest that the effects of microalgae depend on factors such as species, dosage, and animal type or physiological state, warranting further investigation. *Desmodesmus* sp., a protein-rich microalga capable of accumulating high iron concentrations during cultivation, was used to replace portions of soybean meal and corn in diets for anemic weaned piglets [169]. This substitution improved dry matter digestibility and served as an iron source to increase hemoglobin levels and hematocrit, thereby restoring normal growth [169]. Additionally, the cell walls of eukaryotic microalgae primarily consist of indigestible polysaccharides, limiting nutrient bioavailability in monogastric animals. To address this, exogenous feed enzymes can be employed to break down microalgal cell walls, enhancing nutrient digestibility and absorption [170]. Meanwhile, this shift from low-dose supplementation to high-level substitution demands a comprehensive assessment of factors such as digestibility, safety, and animal tolerance, alongside robust evaluation of how these uncertainties affect overall production performance and animal well-being.

Microalgae contain bioactive compounds such as polysaccharides and carotenoids, which exhibit probiotic properties and may positively influence animal immune systems. These components show potential as alternatives to antibiotics or antibiotic-resistant microorganisms. Dietary supplementation with 5% *Chlorella vulgaris* has been shown to enhance antioxidant ability, immune response and intestinal health in piglets without compromising growth performance [171, 172]. Similarly, *S. platensis* could partially replace corn and soybean meal in broiler diets while improving growth performance, meat quality, immunity, and intestinal barrier function [173, 174]. Moreover, *Schizochytrium* addition containing DHA improved the final weight, antioxidant ability and meat fatty acid composition of Ross 308 broilers [153]. Therefore, microalgae are beneficial for the growth performance, livestock product quality, and overall health of monogastric animals.

The unique rumen digestive system of ruminants confers an enhanced capacity to digest microalgal cell walls. Dietary supplementation with microalgae has demonstrated multiple benefits for ruminants, including improved growth performance, optimized feed conversion efficiency, enhanced serum antioxidant capacity, superior meat quality, and reduced methane emissions [175]. *S. platensis* supplementation increased lamb weight gain and feed intake [160], and *Schizochytrium* sp. inclusion improved body weight in first-calf heifers and enhanced outcomes for their newborns [157]. In dairy cows, *Prototheca moriformis* enhanced fiber digestibility and serum lipid profiles [176]. Calves supplemented with *Schizochytrium* sp. exhibited increased daily weight gain and antioxidant enzyme activity [177]. Microalgae are rich in PUFAs and increase the quality of ruminant products. Research has revealed that 0.5% *C. vulgaris* supplementation increased the levels of α-linolenic acid and n-3 PUFA in fresh lamb meat [165]. Similarly, *Schizochytrium* sp. increased EPA and DHA levels in beef and reduced back fat deposition, enhancing lipid metabolism [156, 178]. In dairy production, microalgae can also increase the nutritional quality of milk from ruminants. Furthermore, *Schizochytrium* sp. improved the fatty acid composition of goat milk and cheese, increasing n-3 fatty acids while reducing the n-6/n-3 ratios and saturated fats [179].

## Challenges and prospects

Microalgae have demonstrated significant potential as sustainable bioproduction platforms for high-value bioactive compounds in the feed industry. Several species have been successfully commercialized for specific metabolites: *H. pluvialis* for astaxanthin production, *D. salina* for β-carotene accumulation, *S. platensis* for phycocyanin synthesis, and *P. tricornutum* for lutein generation. Despite these biochemical capabilities, the microalgae-based feed sector faces critical challenges including high costs, low yields, and limited industrial scalability of microalgae species, resulting in the prolonged market dominance of synthetic analogues. To overcome these limitations, future research should focus on the following strategic directions.

### Improving the microalgae efficiency of biosynthesis and metabolism

The biosynthesis and metabolic modification of microalgae face multiple challenges, primarily including the lack of gene editing tools, insufficient understanding of metabolic networks, and compartmentalization limitations in synthesis regions. In tool development, existing technologies fail to overcome technical bottlenecks such as codon bias and transgenic silencing in model species (*C. reinhardtii*), and there is an urgent need to develop novel synthetic biology tools such as optimized promoters and riboswitches. Regarding metabolic engineering strategies, constructing enzyme complexes [180] and targeting subcellular localization [181] can effectively address the problem of enzyme substrate chelation and improve target compound synthesis efficiency. However, there is still a significant gap in the basic understanding of gene clusters and regulatory elements for active substance synthesis, and the synthetic mechanism of high-value product has not been elucidated, which restricts its industrial application. Future metabolic engineering strategies should integrate machine learning approaches with systematic metabolic network analysis to enable more rational and predictive pathway optimization in microalgae [182].

### Enhancing microalgae utilization in feed

Microalgae demonstrate significant potential as both protein sources and functional additives in animal nutrition. A deep understanding of the interactions among microalgae, animals, and their environment is crucial for developing targeted microalgae-based products and advancing commercial-scale production. To improve utilization efficiency, future research should comprehensively analyze the nutritional and metabolic characteristics of microalgae under different conditions, and study the role and mechanism of microalgae in different animal production applications. To maximize the potential of microalgae in animal husbandry, a systematic optimization approach should be implemented, focusing on: (1) refining feed formulations with microalgae inclusion; (2) establishing precision application protocols (through calibration forms, methods, time, duration, and concentration); (3) investigating growth-stage-specific physiological responses to microalgae supplementation; and (4) assessing impacts on gut microbiota composition and overall breeding performance. This multifaceted strategy will significantly enhance the adoption and efficacy of microalgae-based solutions in livestock production systems.

### Exploring the microalgae biosynthesis in circular agriculture

Microalgae-based biosynthesis has emerged as a promising approach for advancing circular agriculture through efficient resource recovery and waste valorization. The process utilizes wastewater nutrients (N, P, K) while producing valuable biomass and generating reusable water suitable for irrigation, crop cultivation and soil remediation. A comprehensive method has been developed for treating pig farm wastewater using microalgae, in which harvested biomass supplemented pig feed, and the treated wastewater was recycled for pen cleaning [183]. Moreover, this innovative model continues to emerge. Pei and Yu [184] proposed microalgae ecological farms for simultaneous saline soil remediation, carbon sequestration, and sustainable production, while Ali et al. [185] implemented “manure-algae-crop” method, using microalgae as a nutrient carrier for fertilizers to crops. However, due to the significant capital investment, high operating costs, and process complexity associated with microalgae biosynthesis, the large-scale implementation of microalgae in agriculture requires comprehensive economic and environmental assessments, with a focus on biosafety, efficiency, and sustainability.

## Conclusion

This review systematically elaborates on the construction strategy and key nodes in the synthesis pathway of microalgae cell factories, particularly exploring the breakthrough application of CRISPR system in optimizing microalgae feed characteristics and the latest progress of microalgae in husbandry production. Emerging genome editing platforms like CRISPR/Cas9 demonstrate distinct advantages over conventional transgenic methods for engineering microalgae cell factory, positioning them as the preferred technology for industrial-scale feed ingredient production. The sustainable development of microalgae biosynthesis for feed requires key advances including implementing comprehensive life cycle assessment, creating circular production systems that integrate carbon fixation and nutrient recovery from manure, and optimizing synergistic economic-environmental benefits. These strategic approaches will enable efficient microalgae cell factories and sustainable production of functional feed ingredients.

## Data Availability

Not applicable.

## Abbreviations

ALE: Adaptive laboratory evolution
ARTP: Atmospheric and room-temperature plasma
ChyB: β-Carotene hydroxylase
dCas9: Dead Cas9
DHA: Docosahexaenoic acid
EMS: Ethyl methanesulfonate
EPA: Eicosapentaenoic acid
GBP: Glutamyl-tRNA reductase-binding protein
GluTR: Glutamyl-tRNA reductase
gRNAs: Guide RNAs
HDR: Homology-dependent recombination
NHEJ: Non-homologous end joining
NTRC: NADPH-dependent thioredoxin reductase C
PAM: Protospacer adjacent motif
PSY: Phytoene synthase
PUFAs: Polyunsaturated fatty acids
RNAi: RNA interference
RNP: Ribonucleoprotein
sgRNA: Single-guide RNA
TALENs: Transcription activator-like effector nucleases
TFs: Transcription factors
TPP: Thiamine pyrophosphate
TRX: Thioredoxins
UV: Ultraviolet
ZFNs: Zinc finger nucleases
